# Supplementary material for: FAAH Inhibition Restores Early Life Stress-Induced Alterations in PFC microRNAs Associated with Depressive-Like Behavior in Male and Female Rats
Source: Int J Mol Sci. 2022 Dec 17;23(24):16101. doi: 10.3390/ijms232416101 (PMC9782513; doi:10.3390/ijms232416101)
Supplement: Supplementary file 1 [file ijms-23-16101-s001.zip › ijms-1999952-supplementary.pdf]

Supplementary information:

Figure S1:

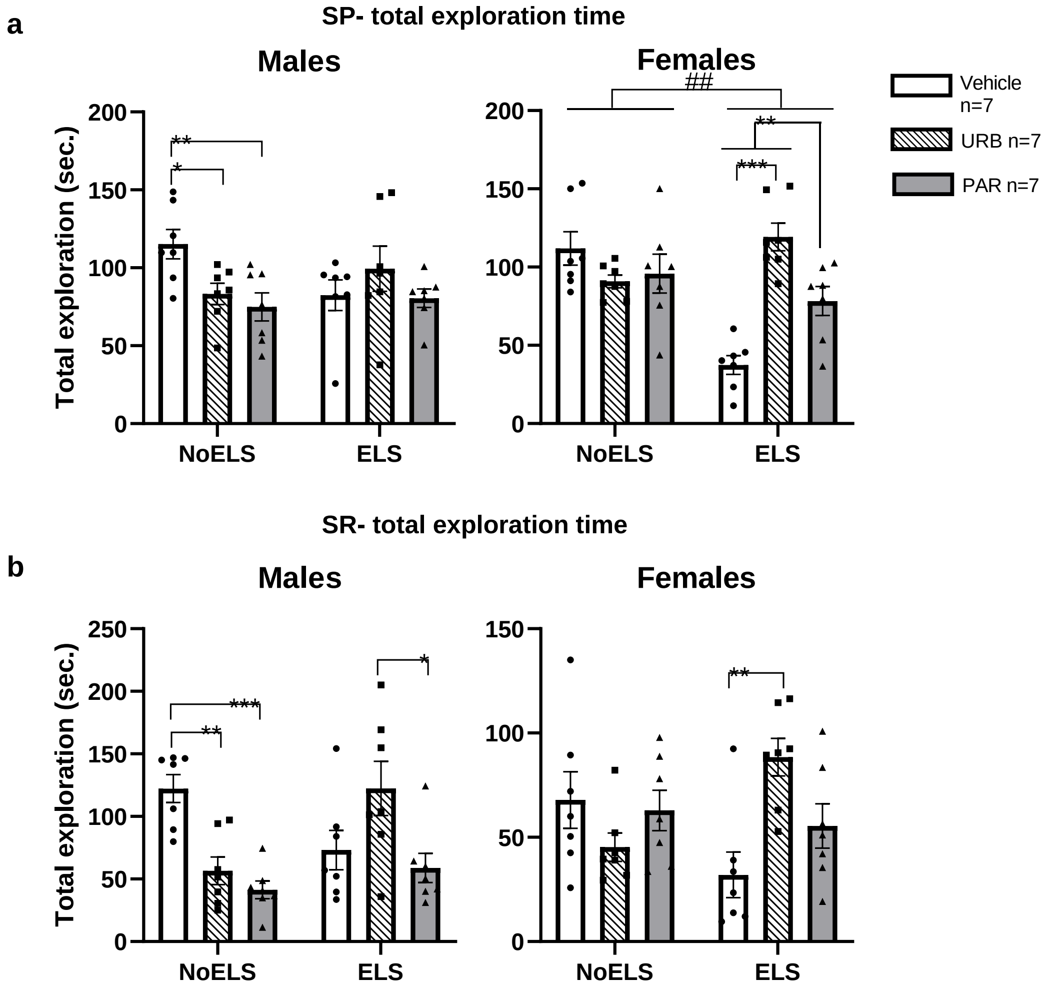

Figure S1. The effects of ELS and chronic late-adolescence treatment with URB or PAR on the total exploration time in the social tests in adult males and females. (a) In the SP test, in NoELS males (left), vehicle treatment increased exploration compared to the URB PAR treatments. In ELS females (right), vehicle treatment decreased exploration compared to the URB and PAR treatments. The PAR treatment decreased the total exploration time compared to the URB treatment. **(b)** In the SR test, in ELS males (left), URB treatment increased exploration compared to PAR treatment. Also, vehicle treatment increased exploration compared to the URB and PAR treatments in NoELS males. In ELS females (right), URB treatment increased the total exploration time compared to vehicle treatment.

ELS: early life stress; PAR: paroxetine; SP: social preference; SR: social recognition; URB: URB597. *, p<0.05; **, p<0.01; ***, p<0.001 indicate statistically significant effects followed by post-hoc comparisons. ##, p<0.01 indicate statistical significance in main effects.

For exploration time in the SP test (**Figure S1a**), univariate ANOVA revealed significant effects of ELS ($F_{(1,72)}$=5.38, p=.023), drug×sex ($F_{(2,72)}$=4.94, p=.010) and ELS×drug ($F_{(2,72)}$=17.01, p<.001) interactions, with no effect of sex ($F_{(1,72)}$=.004, p=.947), drug ($F_{(2,72)}$=3.07, p=.052), ELS×sex ($F_{(1,72)}$=2.67, p=.107) and ELS×drug×sex ($F_{(2,72)}$=1.87, p=.161).

In males (**Figure S1a, left**), two-way ANOVA revealed significant effect of ELS×drug interaction ($F_{(2,36)}$=3.58, *p*=.038), with no effect of ELS ($F_{(1,36)}$=0.22, *p*=.642) and drug ($F_{(2,36)}$=2.46, *p*=.090). Post-hoc comparisons revealed that NoELS-Vehicle males demonstrated increased exploration compared to NoELS-URB (*p*=.039) and NoELS-PAR (*p*=.009) groups. This suggests that both URB and PAR had an effect by themselves on exploration time in the SP test in non-stressed males.
In females (**Figure S1a, right**), two-way ANOVA revealed significant effects of ELS ($F_{(1,36)}$=8.41, *p*=.006), drug ($F_{(2,36)}$=5.79, *p*=.007) and ELS×drug interaction ($F_{(2,36)}$=16.5, *p*<.001). Post-hoc comparisons revealed that ELS-URB females demonstrated increased exploration compared to ELS-Vehicle (*p*<.001) and ELS-PAR (*p*=.006) groups, suggesting that URB restored the ELS-induced decrease in total exploration time. Also, the ELS-PAR group demonstrated increased exploration compared to the ELS-Vehicle group (*p*=.006).

For total exploration time in the SR test (**Figure S1b**), univariate ANOVA revealed significant effects of sex ($F_{(1,72)}$=8.41, *p*=.005), drug ($F_{(2,72)}$=4.19, *p*=.019), drug×sex ($F_{(2,72)}$=5.44, *p*=.006) and ELS×drug ($F_{(2,72)}$=15.77, *p*<.001) interactions, with no effect of ELS ($F_{(1,72)}$=0.64, *p*=.424), ELS×sex ($F_{(1,72)}$=.66, *p*=.419), and ELS×drug×sex ($F_{(2,72)}$=.76, *p*=.470).

In males (**Figure S1b, left**), two-way ANOVA revealed significant effect of drug ($F_{(2,36)}$=6.74, *p*=.003) and ELS×drug interaction ($F_{(2,36)}$=8.66, *p*=.001), with no effect of ELS ($F_{(1,36)}$=1.01, *p*=.320). Post-hoc comparisons revealed that ELS-URB males demonstrated increased exploration compared to ELS-PAR (*p*=.040). In the NoELS groups, the NoELS-Vehicle group demonstrated increased exploration compared to the NoELS-URB (*p*=.001) and NoELS-PAR (*p*<.001) groups. This suggests that both URB and PAR had an effect by themselves on total exploration time in the SR test in non-stressed males.
In females (**Figure S1b, right**), two-way ANOVA revealed significant effect of ELS×drug interaction ($F_{(2,36)}$=7.54, *p*=.002), with no effect of ELS ($F_{(1,36)}$=0.00, *p*=.995) and drug ($F_{(2,36)}$=1.35, *p*=.272). Post-hoc comparisons revealed that ELS-URB rats demonstrated increased exploration compared to the ELS-Vehicle group (*p*=.003), suggesting that URB restored the ELS-induced decrease in total exploration time.

Figure S2:

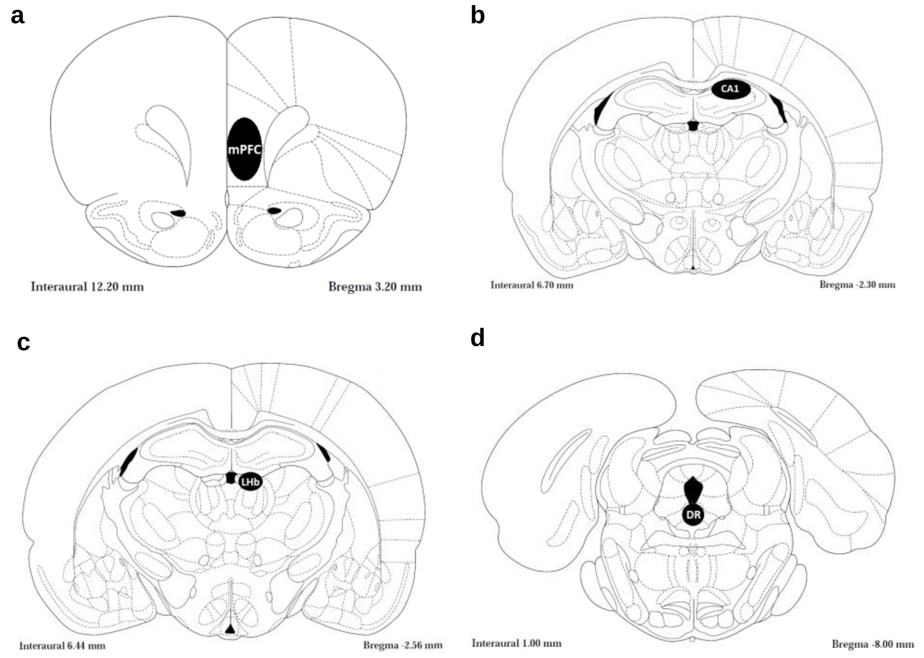

Figure S2. Brain areas for molecular analysis. Atlas illustration in a coronal view; samples were obtained by punches (1 mm diameter). The numbers refer to the distance from Bregma (Paxinos & Watson, 2007): **(a)** mPFC at position 3.20 mm anterior to bregma. **(b)** CA1 at position -2.30 mm posterior to bregma. **(c)** LHb at position -2.56 mm posterior to bregma. **(d)** DR at position -8.00 mm posterior to bregma.

DR: dorsal raphe; LHb: lateral habenula; mPFC: medial prefrontal cortex.
